# Regulatory roles of SP-A and exosomes in pneumonia-induced acute lung and kidney injuries

**DOI:** 10.3389/fimmu.2023.1188023

**Published:** 2023-05-15

**Authors:** Xinghua Chen, Junping Guo, Salma Mahmoud, Gautam Vanga, Tianyi Liu, Wanwen Xu, Yunhe Xiong, Weichuan Xiong, Osama Abdel-Razek, Guirong Wang

**Affiliations:** ^1^ Department of Surgery, SUNY Upstate Medical University, Syracuse, NY, United States; ^2^ Department of Nephrology, Wuhan University, Renmin Hospital, Wuhan, Hubei, China; ^3^ Department of Microbiology and Immunology, SUNY Upstate Medical University, Syracuse, NY, United States

**Keywords:** acute kidney injury (AKI), exosomes, innate immunity, renal tubular epithelial cells, surfactant protein A (SP-A)

## Abstract

**Introduction:**

Pneumonia-induced sepsis can cause multiple organ dysfunction including acute lung and kidney injury (ALI and AKI). Surfactant protein A (SP-A), a critical innate immune molecule, is expressed in the lung and kidney. Extracellular vesicles like exosomes are involved in the processes of pathophysiology. Here we tested one hypothesis that SP-A regulates pneumonia-induced AKI through the modulation of exosomes and cell death.

**Methods:**

Wild-type (WT), SP-A knockout (KO), and humanized SP-A transgenic (hTG, lung-specific SP-A expression) mice were used in this study.

**Results:**

After intratracheal infection with *Pseudomonas aeruginosa*, KO mice showed increased mortality, higher injury scores, more severe inflammation in the lung and kidney, and increased serum TNF-α, IL-1β, and IL-6 levels compared to WT and hTG mice. Infected hTG mice exhibited similar lung injury but more severe kidney injury than infected WT mice. Increased renal tubular apoptosis and pyroptosis in the kidney of KO mice were found when compared with WT and hTG mice. We found that serum exosomes from septic mice cause ALI and AKI through mediating apoptosis and proptosis when mice were injected intravenously. Furthermore, primary proximal tubular epithelial cells isolated from KO mice showed more sensitivity than those from WT mice after exposure to septic serum exosomes.

**Discussion:**

Collectively, SP-A attenuates pneumonia-induced ALI and AKI by regulating inflammation, apoptosis and pyroptosis; serum exosomes are important mediators in the pathogenesis of AKI.

## 1 Introduction

Acute kidney injury (AKI) is the most common complication in septic patients, affecting more than half of the intensive care unit (ICU) patients with high morbidity and mortality rates (1–3). Kidney is one of the most common organs affected by sepsis, causing in sepsis-associated AKI. The annual global incidence of sepsis-induced AKI might be approximately 6 million cases or nearly 1 per 1000 population (4). AKI occurs in 40-50% of septic patients and increases the mortality and morbidity six to eight-fold (5, 6). Sepsis-associated AKI patients have a significantly increased mortality relative to those with AKI of other etiologies (7). Pneumonia is one of the most common reasons inducing sepsis. Our previous studies demonstrated that *Pseudomonas aeruginosa* can induce sepsis and AKI in pneumonia murine model (8, 9).

Understanding the cellular and molecular mechanisms of septic AKI is important for the development of new therapeutic approaches for patients with septic AKI. Recently, several animal models have shown that inflammation, apoptosis, and pyroptosis play a role in the septic AKI (10, 11). AKI itself can regulate the immune response during inflammation; recent evidence indicates that both innate and adaptive immune responses are involved in renal tubular cell damages in AKI and recovery from it (12, 13). Surfactant protein A (SP-A), a member of the C-type lectin family, is primarily expressed in the lung (14), and also in the urinary tract tissues (including the kidney, urethra, bladder, and urethra) (15). We have previously found that septic SP-A/D KO mice suffered from more severe kidney injury and excessive apoptotic cells compared with septic wild-type mice in sepsis-induced AKI (16).

Extracellular vesicles (EVs) are membranous vesicles that contain active proteins, lipids, and several types of genetic materials such as miRNAs, mRNAs, and DNAs related to the characteristics of the originating cells. Small-size EVs, also called exosomes, are vesicles between 30 to 100 nm (17). EVs play a vital role in both the physiological and pathological states, and the evidence from studies also shows that exosomes play a role in the injury and repair of AKI (17, 18). Exosomes have recently emerged as critical cargos that contain multiple mediators critical for the pathogenesis of sepsis-associated organ dysfunctions (19). Cytokines and chemokines play essential roles in the progression of sepsis (20), and Gao et al. (21) found that cytokines/chemokines from blood existed in both the soluble and insoluble exosomes, exosomes enriched with cytokines/chemokines have a critical role in T cell proliferation, differentiation, and chemotaxis during the sepsis process.

In this study, we used wild-type (WT), SP-A knockout (KO) mice, and humanized SP-A transgenic (hTG) mice with lung-specific SP-A expression to study the mechanistic roles of organ-specific SP-A in the pneumonia-induced Acute Lung Injury (ALI) and AKI. We found that the exosomes in the septic mouse serum are one of the major factors to induce Kidney injury, however, the SP-A is a proactive factor against it. Furthermore, the results from *In vivo* study are confirmed by *In vitro* study with primary renal proximal tubular epithelial cells (RTECs) isolated from WT and SP-A KO mice. The results revealed that the pulmonary and renal SP-A have protective roles in sepsis-induced AKI and the exosomes involved in the processes of AKI.

## 2 Results

### 2.1 SP-A expression in the lung and kidney of WT, SP-A KO, and hTG SP-A2 (1A^0^)

**Figure 1:**
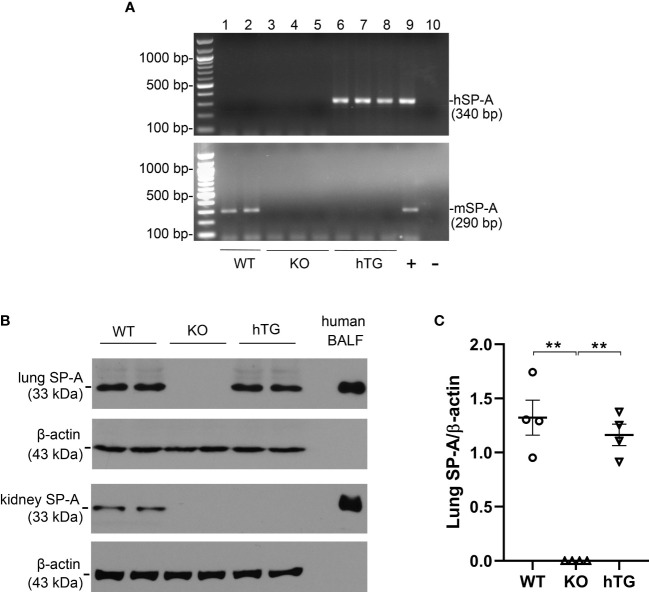
SP-A mRNA and protein expression in KO and hTG mouse. **(A)** Genotyping analysis of WT, KO, hTG mice by PCR. Recombinant plasmid was used as positive control and H_2_O was used instead of DNA template as negative control. The mice were genotyped with primers for hSP-A and mSP-A, respectively. hSP-A PCR product is 340 bp and mSP-A product is 290 bp. hTG mice carry hSP-A gene (No. 6-8), WT mice have mSP-A (No.1 and 2) and KO mice with neither hSP-A nor mSP-A (No.3-5). **(B)** SP-A expression in the lung and kidney tissues from sham WT, KO and hTG mice by Western blotting analysis using an anti-SP-A antibody. Human BALF was used as positive control. SP-A (33 kDa) was expressed in both the lung and kidney of WT mice, in neither the lung nor kidney of KO mice and in the lung but not kidney of hTG mice. **(C)** Quantification of SP-A expression by Western blot analysis in the lung of sham WT, KO, and hTG mice. The data demonstrate similar SP-A level in the lung tissues of sham WT and hTG mice (**P<0.01). t-test (n= 6 mice/group).

Three genotypes of mice (WT, SP-A KO, and hTG SP-A2 (1A^0^)) were used for the study. The hSP-A and mSP-A genotypes of WT, KO, and hTG mice are represented in **Figure 1A**. The expression of SP-A protein was analyzed using Western blotting analysis (**Figure 1B**) against the SP-A antibody. SP-A expression in the lung and kidney of WT mice but only in the lung of hTG mice. No SP-A protein, as expected, was expressed in SP-A KO mice. Quantitative analysis of SP-A expression indicated similar SP-A level in the lungs of WT and hTG mice (**Figure 1C**).

### 2.2 Decreased bacterial clearance and animal survival in infected SP-A KO mice

**Figure 2:**
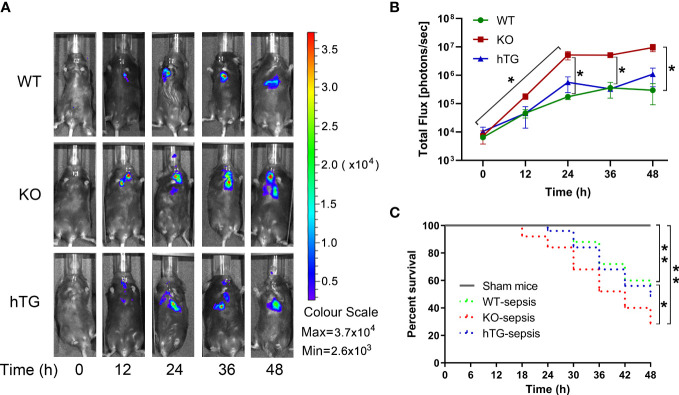
Mice lacking pulmonary SP-A are more susceptible to bacterial infection. **(A)** Representative *In Vivo* bioluminescence imaging of bacteria following intratracheal inoculation of *P. aeruginosa* in WT, KO and hTG mice. After infection, mice were imaged at each time points as listed below. **(B)** The bioluminescence signal in three groups of infected mice progressively increased and peaked at 36-48 h after infection. The KO mice showed higher level of bioluminescence than WT and hTG mice at 24 h after infection and beyond, whereas no significant difference between infected WT and hTG mice. *P< 0.05 *vs*. WT or hTG, t test (n= 6 mice/group). **(C)** The survival curves showed lower survival rate in infected KO mice compared to infected WT, but no difference between infected hTG and WT mice. *P< 0.05 *vs*. WT, Log-rank test (n=25 mice/group).

To examine the role of SP-A in pneumonia-induced sepsis, bacterial dynamic changes were compared in the lungs of WT, KO, and hTG mice at 0, 12, 24, 36, and 48h after intratracheal inoculation of bioluminescent *P. aeruginosa* by *In vivo* imaging method (**Figure 2A**). The results indicated that infected KO mice showed significantly higher levels of bioluminescence than the infected WT and hTG mice at 24 h, 36h and 48h after infection (p<0.05), but no difference was found between infected WT and hTG mice (**Figure 2B**). Furthermore, a higher rate of mortality in infected KO mice was also detected when compared with infected WT (p<0.05), but no difference was found between infected hTG and WT mice (**Figure 2C**).

### 2.3 Decreased SP-A level in the lung and kidney of septic SP-A KO mice

**Figure 3:**
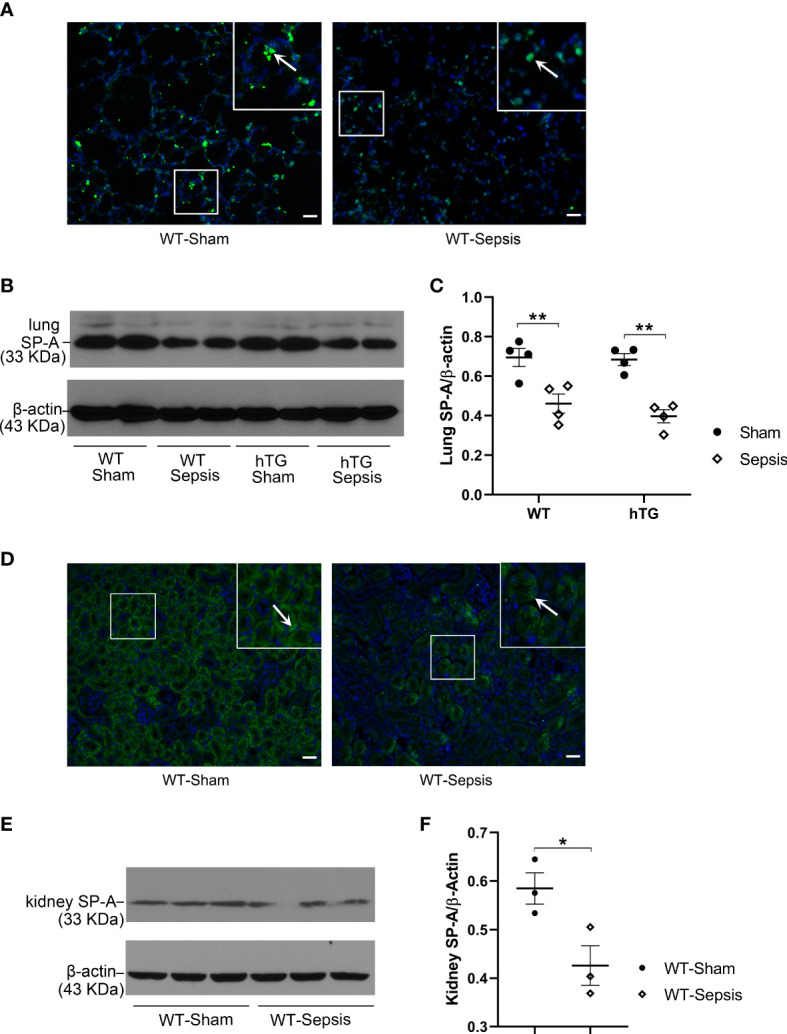
The effect of sepsis on the SP-A expression in the lung and kidney. **(A)** Immunofluorescence staining for SP-A on representative lung sections of WT sham and sepsis mice. SP-A (green color for SP-A, blue color for nuclei) is predominantly expressed in the alveolar epithelial cells. **(B, C)** Western blot analysis of SP-A expression in the lung tissue of WT and hTG mice from sham and sepsis mice. SP-A expression was significantly reduced at 48 h after infection in both sepsis WT and hTG mice. No difference was observed between WT and hTG mice with or without sepsis. *P < 0.05, **P < 0.01, (n= 6 mice/group). **(D)** Immunofluorescence staining for SP-A on representative kidney sections of WT sham and sepsis mice. SP-A is predominantly expressed in the renal tubular epithelial cells. **(E, F)** Western blot analysis of SP-A expression in the kidney tissue of WT mice from sham and sepsis mice. SP-A expression was significantly reduced at 48 h after infection. *P< 0.05, (n=6 mice/group).

SP-A expression in the lung and kidney of infected WT and hTG mice were examined by immunofluorescence (IF) and Western blotting analyses. The results showed that SP-A level in the lung reduced significantly 48 hours after infection in both WT and hTG mice when compared with their respective sham controls, but no difference was found between infected WT and hTG mice 48 hours post-infection (p<0.01) (**Figures 3A–C**). In addition, SP-A expression in the kidney of infected WT mice decreased when compared to sham controls (p<0.05) (**Figures 3D–F**). The IF analysis with the SP-A antibody indicated that the immunoreactivity for SP-A was predominantly present in the proximal tubules of the kidney (**Figure 3D**).

### 2.4 Deteriorated lung injury in septic SP-A KO mice

**Figure 4:**
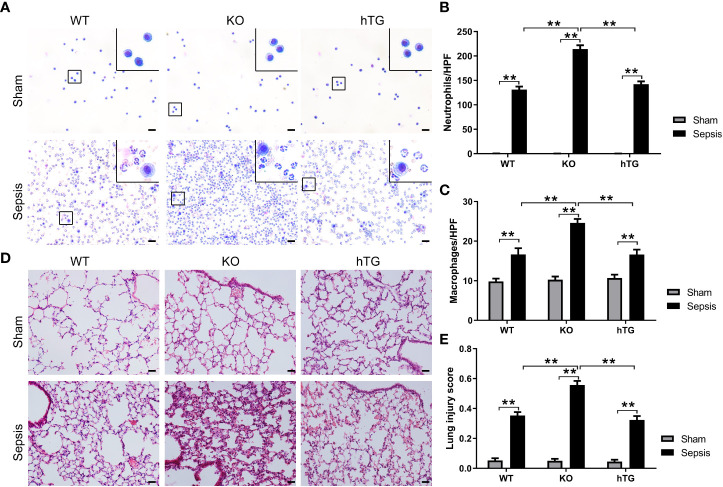
KO mice showed more severe lung injury in bacterial pneumonia compared to WT and hTG mice. **(A)** Representative BALF cytology of each group from sham and infected mice. The cell pellets of BALF were mounted on a slide by the cytospin centrifugation. The slides were stained using the Hema-3 Stain Kit. Morphologically normal macrophages with no neutrophils in sham WT mice and in hTG mice were observed. *P. aeruginosa* infection causes predominant neutrophils in the BALF from three groups of infected mice. Scale bars = 200μm. **(B, C)** Quantification of neutrophils and macrophages in the BALF. Neutrophils and macrophages per slide were counted at ×400 magnification under light microscopy. There was no significant difference between sepsis WT and hTG mice, but the quantification was significantly higher in infected KO mice compared to infected WT and hTG mice (*P*<0.01). **(D)** Representative histological sections of lungs from each group. H&E staining indicates normal lung structures in both sham WT and hTG mice, but occasional slight enlargement of distal airspaces in sham KO mice. *P. aeruginosa* infection induced severe histological lung damage, including diffuse inflammatory cells infiltration in alveoli and interstitial, protein debris accumulation and interstitial edema in infected mice. **(E)** Semi-quantitative histological lung injury score was assessed. There is no significant difference among sham groups. The lung injury score is significantly increased after infection compared to sham mice. There is similar lung injury score between infected WT and hTG mice, but infected KO mice showed higher injury score compared to infected WT and hTG mice. Scale bars = 200 μm; **P< 0.01 (n= 20 mice/group).

Alveolar macrophages were observed the predominant cells in the bronchoalveolar lavage fluid (BALF) of all sham groups. No difference in macrophages was observed in three sham groups of mice but in the BALF from three infected mice groups, the neutrophils were the predominant cells (**Figure 4A**). The numbers of neutrophils (**Figure 4B**) and macrophages (**Figure 4C**) were significantly higher in the BALF from infected KO mice compared to infected WT and hTG mice, but no difference was observed between infected WT and hTG mice (**Figure 4C**). Histological analysis of the lung indicated normal morphology in both sham WT and hTG mice but a slight enlargement of distal airspaces in sham KO mice (**Figure 4D**). *P. aeruginosa* infection induced severe lung injury. The pathological changes include diffuse inflammatory cells infiltration in alveoli and interstitial, protein debris accumulation, and interstitial edema in the lung, which were similar changes in infected WT and hTG mice, but more degree of damages in infected KO mice (**Figure 4D**). The data of lung injury score indicated that infected WT and hTG mice had comparable injury scores, whereas infected SP-A KO mice had higher score of lung injury compared to infected WT and hTG mice (**Figure 4E**).

### 2.5 Increased levels of serum inflammatory cytokines in septic SP-A KO mice

**Figure 5:**
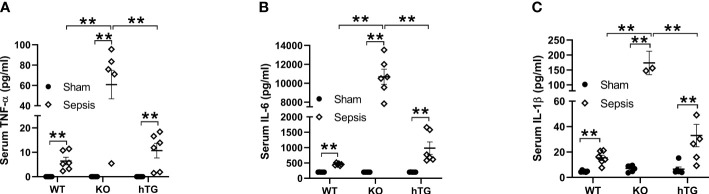
Changes of serum cytokines in pneumonia-induced sepsis. Pro-inflammatory cytokines were determined by ELISA assay for TNF-α **(A)**, IL-6 **(B)** and IL-β **(C)** in the serum of infected WT, KO and hTG mice. The results showed significantly elevated levels of IL-6, TNF-α, and IL-β in all infected mice with an order (KO > WT, KO >hTG) at 48 h after infection, suggesting inhibitory effects of SP-A in the systemic inflammation of pneumonia-induced sepsis. **P< 0.01.

The levels of serum TNF-α, IL-6, and IL-1β were analyzed in both septic and sham mice. The results indicated that the serum TNF-α, IL-6, and IL-1β levels in three septic mice groups were all elevated compared to their respective sham groups (**p<0.01). The levels of TNF-α, IL-6, and IL-1β in septic SP-A KO mice were higher than those of septic WT and hTG mice (**p<0.01) (**Figures 5A–C**).

### 2.6 Increased renal injury in septic SP-A KO mice

**Figure 6:**
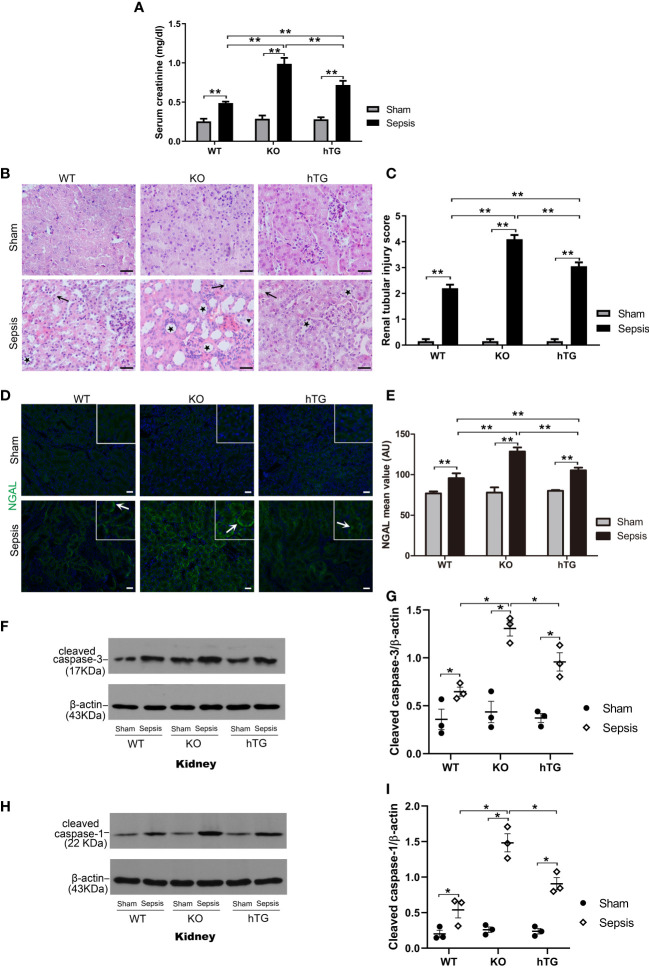
Effects of pulmonary and/or renal SP-A on sepsis-induced AKI. **(A)** Quantitative analysis showed that serum creatinine level was significantly higher in infected KO mice compared to infected WT and hTG mice. Furthermore, when compared to infected WT mice, hTG mice had significantly higher renal injury score. *P< 0.05, **P<0.01, t test (n= 6 mice/group). **(B)** Renal histological analysis by H&E staining showed normal kidney architecture in all sham groups of mice, suggesting that SP-A deficiency in the kidney did not affect kidney development and formation of normal kidney structure. Sepsis induced AKI was characterized by the presence of vacuolar degeneration of tubular cells (arrows), and brush border loss with tubular lumen dilatation (stars). Magnification 400×. Scale bars= 50μm. **(C)** Semi-quantitative analysis demonstrated that renal injury score was significantly higher in infected KO mice compared to infected WT and hTG mice. Furthermore, when compared to infected WT mice, hTG mice had significantly higher renal injury score. *P< 0.05, **P<0.01, t test (n= 6 mice/group). **(D)** Renal NGAL immunofluorescence staining showed sepsis induced AKI was characterized by the presence of NGAL. Magnification 200×. Scale bars= 100 μm. **(E)** Semi-quantitative analysis demonstrated that renal NGAL fluorescence density was significantly higher in infected KO mice compared to infected WT and hTG mice. Furthermore, when compared to infected WT mice, hTG mice had significantly higher renal injury score. *P< 0.05, **P<0.01, t test (n= 6 mice/group). **(F, G)** Western blot analysis demonstrated increased cleaved caspase-3 protein level in infected KO and hTG mice compared to infected WT mice 48 hours after infection, *P< 0.05. **(H, I)** Western blot analysis demonstrated increased cleaved caspase-1 protein level in infected KO and hTG mice compared to infected WT mice 48 hours after infection, *P< 0.05, (n=3 mice/group).

To assess renal injury in septic mice, serum creatinine level was analyzed, and histological changes of kidney sections stained with H/E staining were examined by two experienced pathologists blind to the experimental design. The results showed that serum creatinine levels in septic groups were all higher than in the Sham groups (p<0.01), while septic KO and hTG mice exhibited higher serum creatinine levels compared to septic WT mice. Septic KO mice had the highest serum creatinine level among the three septic mice groups (**Figure 6A**). Histological analysis showed normal kidney architecture in all sham controls, suggesting that renal SP-A deficiency did not result in spontaneous renal injury (**Figure 6B**). However, remarkable pathological changes were observed in the kidney tissues from septic mice (**Figure 6B**). Infected SP-A KO and hTG mice showed more severe kidney damage, which are characterized by tubular degeneration, loss of brush border, and tubular luminal cast formation as well as cell death when compared to infected WT mice (**Figure 6B**). We furtherly analyzed renal injury score, the results showed infected KO mice had higher kidney injury score compared to infected WT and hTG mice; of interesting, septic hTG mice had higher renal injury score than infected WT mice (**Figure 6C**), suggesting lacking pulmonary and/or renal SP-A were more susceptible to sepsis-induced AKI. Furthermore, neutrophil gelatinase-associated lipocalin (NGAL), one positive AKI biomarker, was found to be in higher expression after infection by IF analysis (**Figures 6D, E**). The order of NGAL level is KO>hTG>WT in infected mice 48 hours post-infection (**Figure 6E**).

We further identified cell death types in the kidney of septic mice by analyzing the activation of two biomarkers of apoptosis and pyroptosis, i.e., cleaved caspase-3 (17 kDa) and cleaved caspase-1 (22 kDa) respectively. The results from Western blotting analysis showed that increased both cleaved caspase-3 (17 kDa) and cleaved caspase-1 (22 kDa) levels in the kidneys of septic mice compared to sham mice (p<0.05) (**Figures 6F–I**), indicating that both apoptosis and pyroptosis are involved in the kidney injury. Furthermore, the levels of cleaved caspase-3 and cleaved caspase-1 are higher in infected SP-A KO mice compared to infected WT and hTG mice (**Figures 6G, I**).

### 2.7 Characteristics of serum exosomes from septic and sham mice

**Figure 7:**
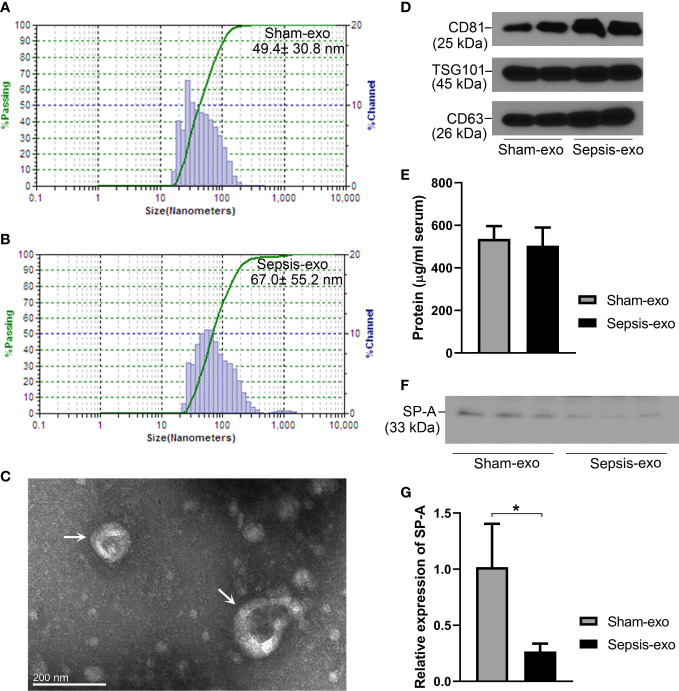
Identification of exosomes and SP-A expression in exosomes in septic and sham mice. **(A, B)** Characterization of exosomes sizes. **(C)** Exosomes particles were verified by electron microscope. **(D)** Western blotting analysis of exosome surface markers (CD81, TSG101, and CD63) expression. **(E)** Protein concentration of serum-derived exosomes. **(F)** Western blotting analysis of SP-A protein expression in exosomes in sham and septic mice. **(G)** Quantitative analysis of SP-A protein expression in exosomes. *P<0.05 (n=3 mice/group).

Exosomes from septic and sham mice serum were isolated and identified by several methods. As shown in **Figures 7A, B**, the particle size ranged between 15 nm and 150 nm, and the principle peak sizes of the particles were 49.4 and 67.0 nm from sham and septic mice, respectively. Exosome particles were further verified by electron microscopy (**Figure 7C**). The presence of the exosome markers CD81 (tumor susceptibility gene), TSG101and CD63 was confirmed in both the sham and sepsis samples by Western blotting analysis (**Figure 7D**), indicating the isolated particles were exosomes. We found the protein concentrations of serum exosomes are similar between sham and septic mice (**Figure 7E**). Moreover, we detected SP-A protein in serum exosomes derived from septic and sham mice, but the SP-A level was lower (p<0.05) in the septic serum exosomes compared to the sham serum exosomes (**Figures 7F, G**).

### 2.8 Exosomes derived from septic mouse serum induced mouse RTECs apoptosis and pyroptosis *in vitro*

**Figure 8:**
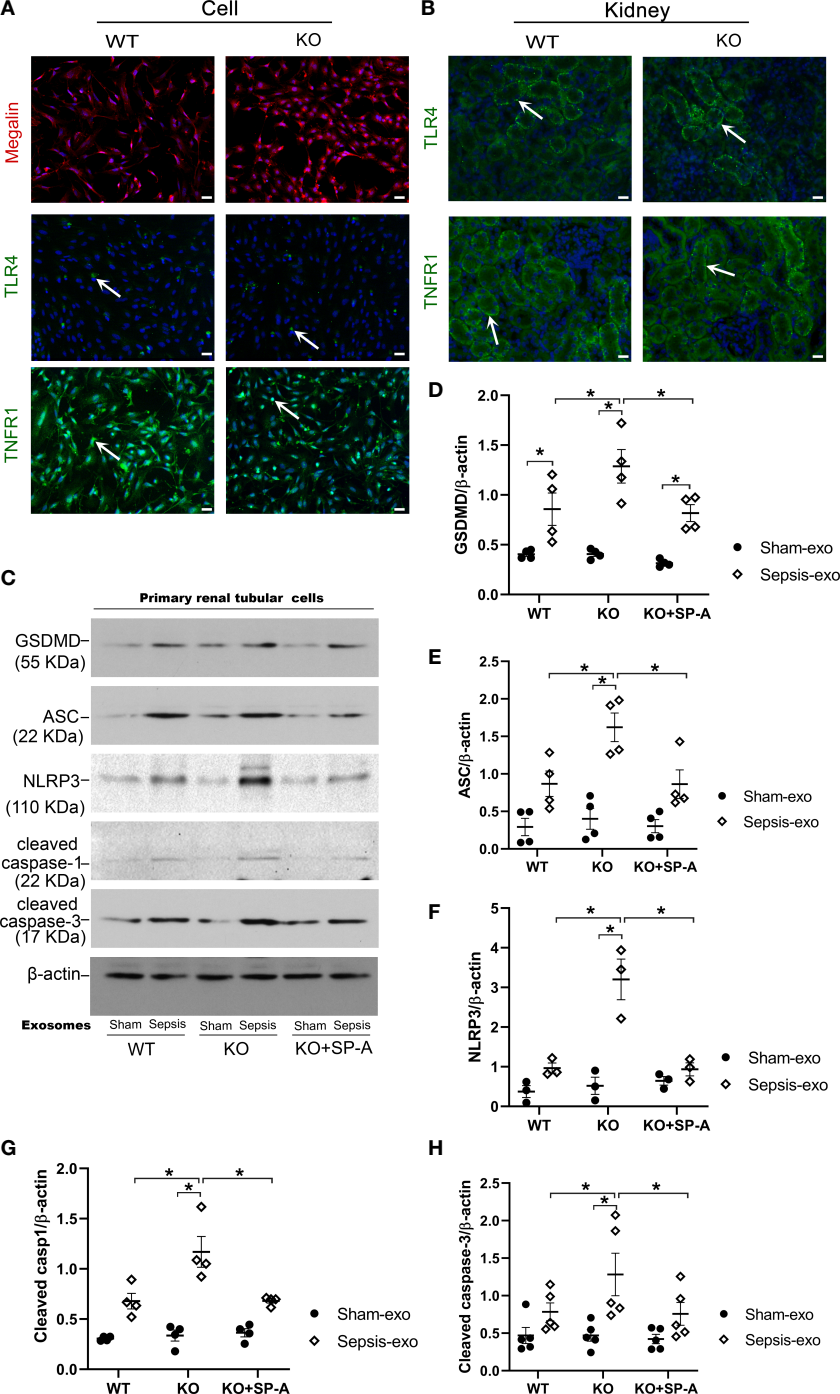
Exosomes from septic mice induced RTECs apoptosis and pyroptosis. **(A)** IF staining of RTECs from WT and KO by Megalin, TLR4 AND TNFR1. More than 95% of isolated cells from WT mice showed Megalin positive. And about 50% of cells showed TLR4 positive, 90% of cells showed TNFR1 positive. Magnification 200×. Scale bars= 100μm. **(B)** IF staining of Kidney tissues from WT and KO mice by TLR4 and TNFR1, about 50% of renal proximal tubular cells showed TLR4 positive, and more than 50% of renal proximal tubular cells showed TNFR1 positive. **(C)** Western blot demonstrated different levels of cleaved caspase-3, cleaved caspase-1, ASC, NLRP3, GSDMD, proteins in sham serum exosomes and sepsis serum exosomes treated WT and SP-A KO RTECs with or without SP-A protein (10 µg/ml) after 24h. **(D–H)** Western blot semi-quantitative analysis showed increased levels of cleaved caspase-3, cleaved caspase-1, ASC, NLRP3, and GSDMD proteins in sepsis serum exosomes treated group, compared to the sham serum treated group, *P< 0.05, (n=3 mice/group).

To explore the mechanistic role of SP-A on the renal tubular injury, RTECs from the kidneys of WT and KO mice were isolated and used for *In vitro* study. The isolated primary cells from WT and KO mice were confirmed as RTECs by IF staining analysis for Megalin, a proximal tubular-specific biomarker. The results showed that more than 95% of the isolated cells were positive for Megalin (PTEC biomarker) (**Figure 8A**), suggesting that these primary isolated cells contain more than 95% of RTECs, which are appropriate for the following study. We further identified RTECs from S1 and S2 segments of the proximal tubule with TLR4 and TNFR1 biomarkers, respectively (22, 23). The results showed about 10% positive cells for TLR4 and about 90% positive cells in RTECs for TNFR1 (**Figure 8A**). The TLR4 and TNFR1 expression was also detected in renal proximal tubular cells (**Figure 8B**).

Primary RTECs from WT and KO mice were exposed to 50 µg/ml of exosomes from either septic or sham mouse serum in serum-free medium for 24 hours. Several biomarkers of apoptosis and pyroptosis were examined in treated RTECs by Western blotting. The results showed that exosomes derived from septic mouse serum could significantly increase apoptotic (cleaved caspase-3) and pyroptotic (cleaved caspase-1, NLRP3, ASC, GSDMD) markers expression in treated KO RTECs compared to the exosomes derived from sham serum (**Figures 8C–H**). Furthermore, the RTECs from WT mice exhibited lower levels of apoptotic and pyroptotic biomarkers compared to the RTECs from KO mice after septic exosome treatment (**Figures 8C–H**). Of interest, RTECs from KO mice exhibited lower levels of the markers of apoptosis and pyroptosis in the presence of SP-A protein (**Figures 8C–H**). These data indicate that septic serum exosomes induced RTECs death by both apoptotic and pyroptotic mechanisms, but SP-A could inhibit exosome-induced RTECs apoptosis and pyroptosis.

### 2.9 Exosomes from septic mouse serum could induce kidney injury *In vivo*

**Figure 9:**
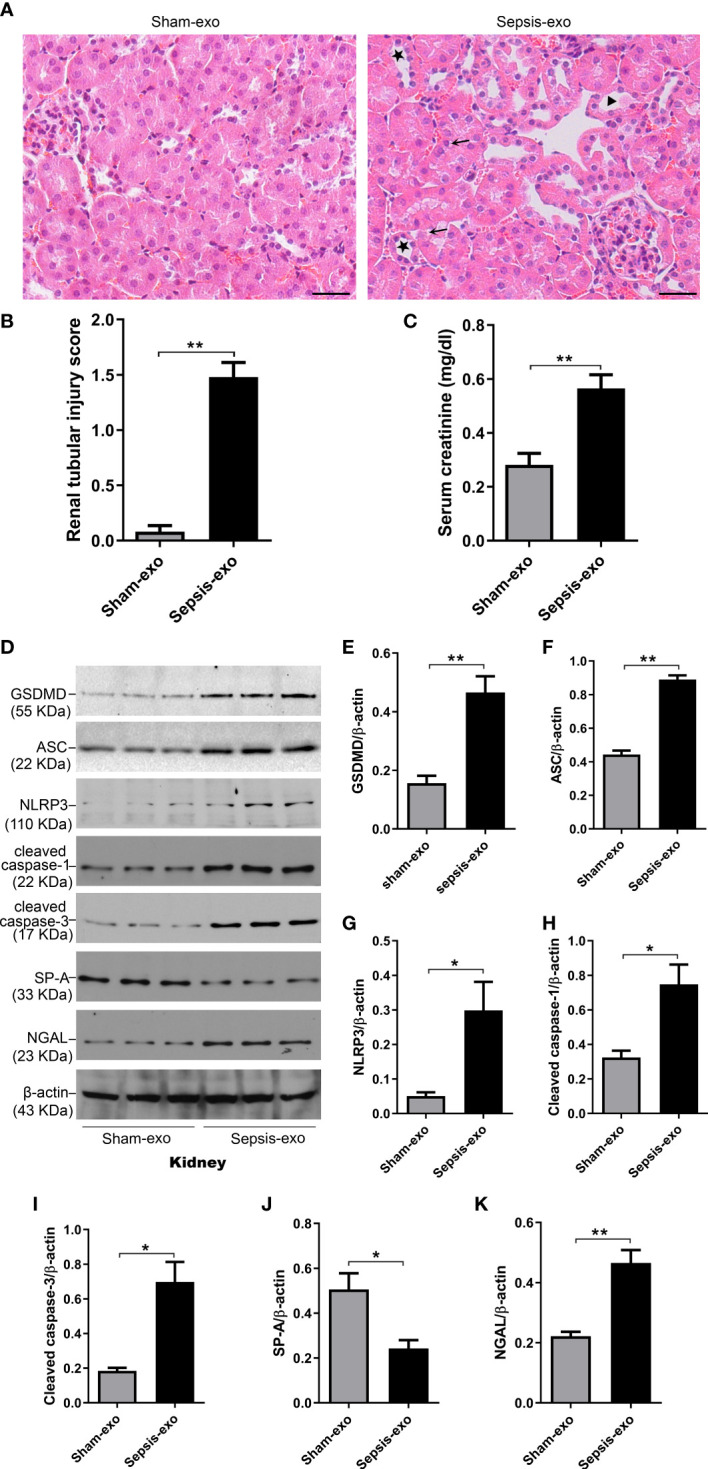
Septic serum exosomes-induced mouse kidney injury. Exosomes from septic mice induced kidney injury **(A)** Representative histological sections of kidneys from sham and septic exosomes groups. H&E staining indicates normal kidney structures in sham exosomes treated WT mice, but injection of septic exosomes causes obvious renal tubular histological damage, including vacuolar degeneration of tubular cells (arrows), and brush border loss with tubular lumen dilatation (stars). **(B)** Semi-quantitative renal tubular injury score was assessed. The renal tubular injury score is significantly increased after septic exosomes injection compared to sham exosomes injected mice. **P< 0.01 (n = 5 mice/group). **(C)** Septic serum exosomes induced mouse serum creatinine changes. It showed that serum creatinine level was significantly higher in septic serum exosomes treated WT mice compared to sham exosomes treated WT mice. **P< 0.01, t test (n= 6 mice/group). **(D)** Western blot demonstrated different levels of cleaved caspase-3, cleaved caspase-1, ASC, NLRP3, GSDMD, and SP-A protein in sham and septic serum exosomes treated mice after 48h. **(E–K)** Western blot semi-quantitative analysis showed increased levels of cleaved caspase-3, cleaved caspase-1, ASC, NLRP3, GSDMD protein, and decreased SP-A protein level in septic serum exosomes treated group, compared to the sham serum treated group, *P< 0.05, **P< 0.01, (n=3 mice/group).

We examined the pathological effect of septic or sham mouse serum exosomes using *In vivo* mouse model with injecting exosomes intravenously. The septic serum exosomes (2 mg of exosome protein/mouse) could induce mouse AKI 48 hours post-injection, as evidenced by the presence of renal tubular injury, such as vacuolar degeneration of tubular cells, brush border loss, tubular lumen dilatation and cast formation (**Figures 9A, B**), and by the increase creatinine level (p<0.05) (**Figure 9C**). Molecular analysis revealed that septic serum exosomes induced increased NGAL (a biomarker of kidney injury), cleaved caspase-3 (apoptosis), cleaved caspase-1, NLRP3, ASC, and GSDMD (pyroptosis) levels in the kidney, compared with sham serum exosomes (p<0.05) (**Figures 9D–K**). SP-A expression was decreased in the kidneys of mice with septic serum exosome treatment, compared to those sham serum exosomes (p<0.05) (**Figure 9J**).

### 2.10 Exosomes from septic mouse serum could induce lung injury *in vivo*

**Figure 10:**
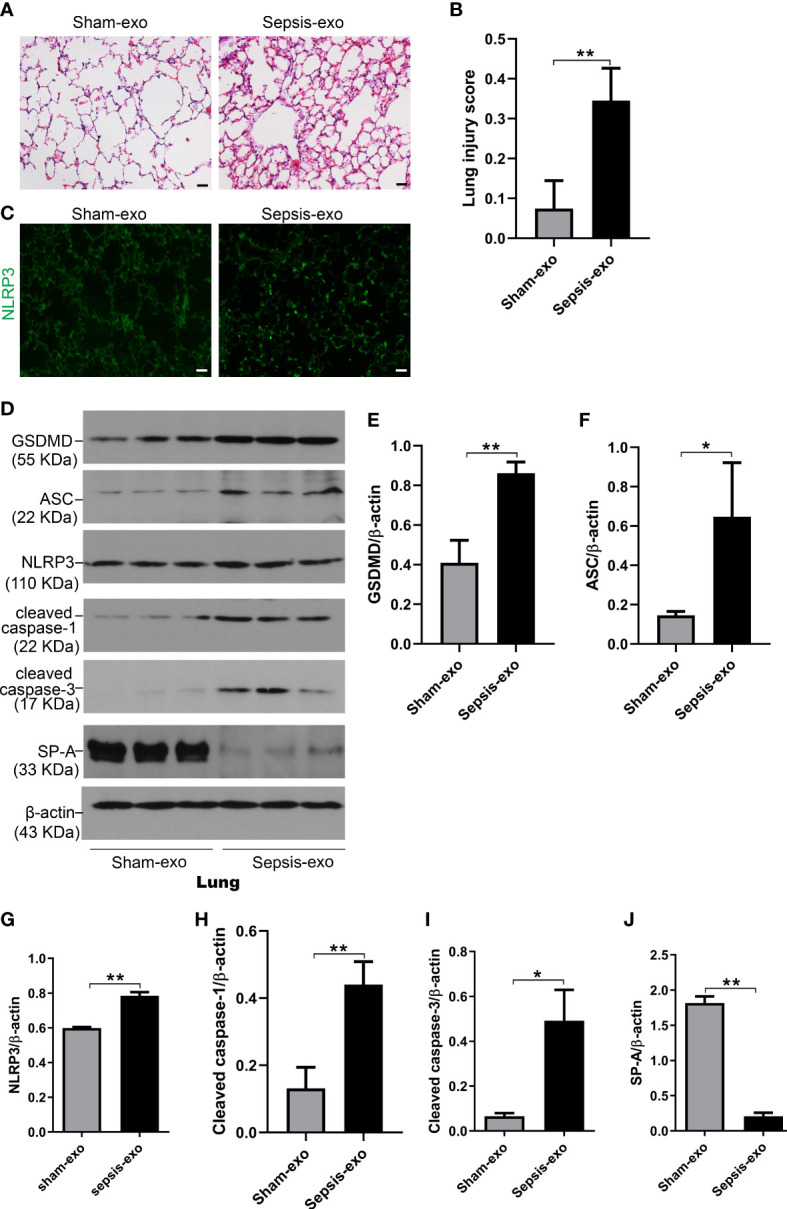
Septic serum exosomes induced mouse lung injury. Exosomes from septic mice induced lung injury **(A)** Representative histological sections of lungs from sham and septic exosomes groups. H&E staining indicates normal lung structures in sham exosomes-treated WT mice, but injection of septic exosomes causes obvious lung histological damage, including inflammatory cells infiltration in interstitial, and alveolar septal thickening. **(B)** Semi-quantitative histological lung injury score was assessed. The lung injury score is significantly increased after septic exosomes injection compared to sham exosomes injected mice. **P< 0.01 (n=5 mice/group). **(C)** The immunofluorescence results showed the NLRP3 expression in septic exosomes injected groups is increased, compared to sham group exosomes injected mice. **(D)** Western blot demonstrated different levels of cleaved caspase-3, cleaved caspase-1, ASC, NLRP3, GSDMD, and SP-A protein in sham and septic serum exosomes treated mice after 48h. **(E–J)** Western blot semi-quantitative analysis showed increased levels of cleaved caspase-3, cleaved caspase-1, ASC, NLRP3, and GSDMD protein, and decreased SP-A protein level in sepsis serum exosomes treated group, compared to the sham serum treated group, *P< 0.05, **P< 0.01 (n=3 mice/group).

We also assessed the effect of the exosomes derived from septic mouse serum or sham mouse serum (control) on lung injury *In vivo* mouse model. As shown in **Figure 10A**, lung histology was almost normal in the control group (exosomes from sham serum), but obvious histopathological changes were observed in the treated group with septic mouse serum-derived exosomes, including neutrophils in the interstitial space and thicker alveolar septa. Analysis of lung injury score demonstrated that the mice injected with septic mouse serum-derived exosomes had significantly higher lung injury scores compared to the control group with sham mouse serum-derived exosomes (p<0.01) (**Figure 10B**). The IF results showed that the NLRP3 expression in the lung of the treated group with septic serum exosomes injected groups is significantly increased, compared to the control group (**Figure 10C**). Molecular analyses revealed that the exosomes derived from septic mouse serum could increase the levels of cell death biomarkers, i.e. cleaved caspase-3 (apoptosis), cleaved caspase-1, and NLRP3, ASC, and GSDMD (pyroptosis) in the lung compared to sham serum exosomes (control) (p<0.01) (**Figure 10D–I**). SP-A expression was decreased in the lung of treated mice with septic serum exosomes compared to sham serum exosomes (control) (p<0.01) (**Figure 10J**). These data indicated that exosomes from septic mouse serum could induce mouse lung injury through a mechanism mediating apoptosis and pyroptosis.

## 3 Discussion

To explore the regulating role of SP-A in the sepsis-induced AKI, we used a bacterial pneumonia-induced sepsis model with three types of mice i.e., WT, SP-A KO, and hTG mice with lung-specific SP-A expression. We found that lack of SP-A in the lung and/or kidney promotes severe lung injury, renal injury, tubular cell apoptosis, pyroptosis, and inflammation in response to pneumonia and sepsis *In vivo*. We also demonstrated that SP-A-deficient RTECs are more susceptible to apoptosis and pyroptosis after treatment with septic serum-derived exosomes compared to treatment with sham serum-derived exosomes.

Severe pneumonia can develop into sepsis (24). *P. aeruginosa* is one of the most common cause of healthcare-associated infection, and in a head-to-head comparison of bloodstream infections (bacteremia), *P. aeruginosa* is associated with higher mortality than other bacteria (25). We devised the pneumonia and sepsis model by intratracheal injection of *P. aeruginosa (* 9). With the development of sepsis, the levels of various inflammatory cytokines in the blood are significantly elevated (26, 27). In this study, we detected the increased presence of serum cytokines IL-6, TNF-α, and IL-1β after the infection. This pneumonia model used induce severe bacteremia, significant septic symptoms and around 50% mortality of infected mice and remarkable kidney injury at 48h after infection. Histological and cellular analysis indicated that sepsis-induced AKI manifested as acute tubular lesions such as brush border loss, tubular epithelial cell death, and cast formation. NGAL has been extensively investigated in various AKI phenotypes, which is released by activated neutrophils and various epithelial cells, including RTECs (4). In previous studies, NGAL showed good sensitivity for the indication of AKI (28). In this study, we found that kidney NGAL expression was upregulated, and the serum creatinine levels were increased, which indicated that AKI occurred after sepsis.

SP-A provides first-line host defense, surfactant stability, and lung homeostasis by binding surfactant phospholipids, pathogens, alveolar macrophages, and epithelial cells. Non-primates have one SP-A gene, whereas humans and primates express two functional SP-A1 and SP-A2 peptides with core intra- and inter-species differences in the collagen-like domain (29). The data from this study indicated that despite having decreased pulmonary SP-A level 48 hours after infection with *P. aeruginosa*, both WT and hTG mice showed increased clearance of bacteria, decreased pulmonary inflammation, and lower lung injury scores compared with infected SP-A KO mice, and sepsis can down-regulated lung SP-A expression. However, hTG mice carrying and expressing human SP-A gene cleared bacteria as efficiently as WT mice in their lungs, where they had similar levels of SP-A expression, thus resulting in decreased levels of lung injury compared to SP-A KO mice. These results indicated that the decreased clearance of *P. aeruginosa* and increase in lung injury observed in the SP-A KO mice is due to the absence of pulmonary SP-A, suggesting that SP-A plays a critical role in the innate host defense and bacteria clearance (30). The survival rate in SP-A KO mice after the infection was, therefore, significantly decreased when compared with WT mice. Mikerov et al. (31) also reported that SP-A KO mice were more susceptible to pneumonia than wild-type mice. Since interactions between SP-A and TLR4 play critical roles in host defense, SP-A peptide is able to control *Pseudomonas aeruginosa* lung infection (32); the therapeutic administration of SP-A peptide reduces the bacterial burden, inflammatory cytokines and chemokines production, lung edema, and tissue damage in *P. aeruginosa*-infected mice (32).

Renal tubules of the kidney play an essential physiological role but are also vulnerable to a variety of injuries like hypoxia, proteinuria, toxins, metabolic disorders, and senescence (33). And renal tubular epithelial damage has long been noted to be the major pathological event in AKI (34). In this study, we observed that sepsis-induced kidney SP-A expression is down-regulated, and infected KO mice showed a significantly severe kidney lesion, especially renal tubules injury, compared to the infected WT and hTG mice. In the previous studies from the mouse unilateral urethra obstruction model, SP-A deficiency aggravated kidney structural damage, macrophage accumulation, and tubulointerstitial fibrosis (35). Renal biopsy in patients with sepsis showed tubular necrosis and epithelial cell apoptosis; the cultured tubular cells, when treated with plasma from sepsis patients, undergo more apoptosis (36). We also found more severe renal tubular cell apoptosis in sepsis groups, and more severe apoptosis in the KO group, compared to the apoptosis in the WT group. Pyroptosis and apoptosis are two types of programmed mechanisms of cell death; but pyroptosis results in cell lysis and release of pro-inflammatory cytokines, including interleukin (IL)-1β and IL-18, into the extracellular space (37, 38). Ye et al. (39) reported that pyroptosis of renal tubular epithelial cells is a key event during septic AKI, and Wang et al. (10) revealed that pyroptosis of renal tubular epithelial cells is a major cause of septic AKI in the zebrafish crispant *In vivo* analysis model. Miao et al. (40) found that tubule cell pyroptosis plays a significant role in initiating tubular cell damage and renal functional deterioration in acute kidney injury. We also found that caspase-1 induced pyroptosis and inflammation in sepsis-induced AKI in this study. The KO group showed more severe renal tubular cell pyroptosis than the WT group.

Extracellular vesicles (EVs) contain active proteins, lipids, and several types of genetic materials such as miRNAs, mRNAs, and DNAs related to the characteristics of their originating cells and play a vital role in both physiological and pathological conditions. Small-size EVs, also called exosomes, are vesicles between 30 to 100 nm (17), and exosomes play a role in the injury and repair of AKI (17, 18). To study the role of exosomes derived from septic mouse serum in AKI and acute lung injury, and the effect of SP-A with regards to the exosomes’ function, exogenous exosomes were intravenously injected into WT mice. We found that septic mouse serum-derived exosomes can induce mouse AKI and acute lung injury. Park et al. (41) observed that nano sized EVs from feces have the capacity to induce local and systemic inflammation when they are introduced into the peritoneum, and thus they concluded that bacterial EVs in feces might contribute partly to the pathology of sepsis. Of interesting, Gao et al. (21) found that pre-administration of exosomes from septic mice can suppressed cytokine production and alleviated tissue injury and also prolonged the survival of Cecal-ligation puncture (CLP) mice. They use a relatively lower dose of exosomes (100 µg/mouse); in this study, we use exosomes in a higher dose (1 mg/mouse), from nearly 2 ml septic mouse serum. Perhaps, different doses of exosomes from the septic mouse serum can induce a different effect. Sepsis-induced AKI may be partly due to the serum exosomes; the role of exosomes may be the important mechanism between different organ injuries as observed previously in a study, plasma-derived exosomes contributed to pancreatitis-associated lung injury (42). We also found that septic mouse serum-derived exosomes can induce mouse kidney inflammatory molecule NLRP3, apoptosis, and pyroptosis markers up-regulation. Kim et al. (43) also found that hypoxia induced a significant increase of NLRP3 in the kidney, and in response to the unilateral ureteral obstruction (UUO), NLRP3 KO mice showed less fibrosis and apoptosis in renal tubular cells than WT mice. Gambim et al. (44) showed that in sepsis, platelet-derived exosomes induced endothelial cell caspase-3 activation and apoptosis through peroxynitrite generation. The serum-derived exosomes could be released from multiple cells such as platelets, leukocytes, endothelial cells, and other cells under both physiological or pathological conditions (45). Therefore, we speculate that the origin of exosomes in the animals with sepsis in the present study is a mixture of several cells, including neutrophils, macrophages, lymphocytes, endothelial cells. It is interesting to identify the exosome-specific component(s) and their origins that induce cell death and activate relevant cell-death molecular signaling pathways in our future study.

At the same time, we found that septic mouse serum-derived exosomes can induce both mouse kidney and lung SP-A down-regulation, and SP-A was expressed in exosomes, the SP-A expression was different in sham group mouse serum-derived exosomes and septic serum-derived exosomes. So, we can speculate that SP-A may play a protective function in exosomes and exosomes-treated mice. It is unclear how SP-A is involved in the modulations of sepsis-induced cell death and organ injury/dysfunction i.e. AKI. SP-A may play its protective effects through several different levels such as interactions with pathogens, regulating inflammatory cells as well as modulating inflammatory mediators like exosomes. To explore the role of SP-A and exosomes in the sepsis-induced AKI, we used exosomes to treat WT and KO primary renal tubular epithelial cells (RTECs), and we found that the septic serum-derived exosomes can induce the RTECs apoptosis, pyroptosis, and SP-A down-regulation, and the cell damage was more serious in the KO group. When SP-A protein was pre-added to RTECs, SP-A protein can attenuate the RTECs injury by septic serum exosomes treatment.

This study has some limitations. First, we observed the cell apoptosis and pyroptosis, and we didn’t evaluate cell death types by the proportion in the kidney. Thus, further studies are needed to explore which cell damage is principal. Secondly, we observed the exosome function in this experimental sepsis, but we didn’t explore which content in exosomes would play a role in mediating AKI. Therefore, it is necessary to continue to explore the molecular mechanisms of exosomes on the cell death of AKI in experimental sepsis and *in vitro* RTECs.

In summary, in this study we found that renal tubular death (apoptosis and pyroptosis), and inflammatory responses are important mechanisms in the sepsis-induced AKI, in which both pulmonary and renal SP-A involved in the lung-kidney crosstalk and directly or indirectly modulate lung and kidney inflammatory responses and renal tubular apoptosis, pyroptosis. These findings indicated that both pulmonary and renal SP-A protein are important for regulating cellular and molecular signaling and pathogenesis of bacterial pneumonia-induced AKI. Therefore, based on previous and present discoveries, SP-A protein may be an interesting component in the exogenous surfactant replacement therapy in clinical sepsis and AKI.

## 4 Materials and methods

### 4.1 Animals

The original SP-A KO mice (C57BL/6 background) used were obtained courtesy of Dr. Hawgood (University of California, San Francisco), and C57BL/6 WT mice were obtained from Jackson Laboratories (Bar Harbor, ME). The hTG SP-A mice carrying hSP-A2-1A^0^ allele without mouse SP-A gene background were generated in our previous works (46). The hTG mice expressed SP-A in the lung but not in kidney. There were no significant phenotypic differences among SP-A KO, hTG SP-A, and matched WT mice. The WT, SP-A KO, and hTG SP-A mice were divided into two groups: the pneumonia/sepsis group (infected with *P. aeruginosa* Xen5 strain) and the control group (the sham group with same surgery and the same volume of the sterile vehicle given). The mice used for this study were bred and kept in the animal core facility at SUNY Upstate Medical University. Mice were housed in specific pathogen-free conditions in a temperature-controlled room at 22°C. The mice used in this study were 8 to 10-week-old male and female mice and experiments were approved by the Institutional Animal Care and Use Committee of the SUNY Upstate Medical University with protocol #380. Additionally, they were performed in line with the National Institute of Health and ARRIVE guidelines on the use of laboratory animals.

### 4.2 *P. aeruginosa*-induced pneumonia and sepsis model

In the initial stage of the project, we performed pilot experiments using different doses of *P. aeruginosa Xen5* in KO and WT mice to determine appropriate doses of bacteria for use in the experimental sepsis model. We found that A dosage of 1×10^5^ CFU/mouse in 50μl of bioluminescent *P. aeruginosa Xen5* (a higher toxic strain) bacterial solution was suitable for generating an optimal bioluminescent signal in the lungs for detection by the *in-vivo* imaging system as well as showing significant characteristics of septicemia. These include a remarkable mortality (about 40-60%) 48 h after infection, severe bacteremia as well as multiple organ injury e.g. ALI and AKI. Consequently, all experiments in the study were performed at a dosage of 1×10^5^ CFU/50μl/mouse via intratracheal inoculation to induce pneumonial sepsis. In brief, mice were anesthetized with intraperitoneal ketamine/xylazine (90 mg/kg ketamine, 10 mg/kg xylazine) injection, which was followed by a 0.5 cm midline neck incision to expose the trachea. Bacterial solution was intratracheally inoculated into the lungs of mice in pneumonia/sepsis group, while 50μl of sterile saline was used for the sham group as a control. At 48 h post-infection, the mice were anesthetized to obtain blood samples, then sacrificed for BALF, lungs, and kidneys, which were harvested for further analyses.

### 4.3 *In vivo* bioluminescence imaging of *P. aeruginosa* after infection

Three types of infected mice were monitored post-infection for 48 hours. Mice were then anesthetized with 2.0% isoflurane to acquire a whole-body image after an exposure to 3 min via an *in vivo* Imaging System (IVIS-200, Caliper Life Sciences, Hopkinton, MA). Additionally, the bioluminescence signal from infected mice was measured at various time points like 0, 12 h, 24 h, 36 h, and 48 h after infection. The signal of bioluminescence was quantified using Living Image software, version 4.1 (Caliper Life Sciences). Data are shown as physical units of radiance in photons/sec per cm^2^ per steradian.

### 4.4 Cytology analysis in BALF

As previously described, the lungs of each mouse were lavaged with 3×0.5 ml of sterile saline. The BALF was then centrifuged for 10 min at 250×g. The pellet was resuspended with 1 ml of sterile saline, and cells in the 0.2 ml resuspended fluid were mounted onto a slide by cytospin centrifuge (Hettich ROTOFIX32 A) at 1000 rpm for 3 min. After that, slides were air-dried and stained with Hema-3 Stain Kit (Fisher Scientific Company, Kalamazoo, MI). The cells were examined with a Nikon Eclipse TE2000-U research microscope (Nikon, Melville, NY).

### 4.5 Kidney functional analysis

Blood samples were collected and then centrifuged at 3,000 rpm for 15 min at 4°C to obtain serum. Serum creatinine level was determined by a commercial assay kit (Thermo Scientific, Middletown, VA) as described previously (8).

### 4.6 Kidney and lung H&E staining

Tracheal injection of 0.5mL of neutral formalin was performed to ensure the inflation fixation of the lungs. The fixed lung and kidney tissues were then embedded in paraffin as previously described (8). Lung and kidney tissues were cut into 5μm and 4μm sections respectively and mounted into the slides, which are then manually stained with Hematoxylin and Eosin (H&E) staining. Histopathology was evaluated by two independent pathologists who did not know the experimental design. Lung injury was evaluated with a 0-2 scale, while kidney injury was semi-quantified by identifying the percent of tubules displaying tubule dilation, cast formation, loss of brush border, and cell necrosis as follows: 0 = none, 1 <10%, 2 = 11-25%, 3 = 26-45%, 4 = 46-75%, and 5 = >76%, as previously stated (8).

### 4.7 Immunofluorescence

Paraffin-embedded lung and kidney tissue sections were deparaffinized, and then immersed in 0.2% Triton X-100 for 45 min as described previously (8). Following blocking with 10% donkey serum (ab7475, Abcam Inc, Cambridge, MA) in PBS for 1 h, slides were immunostained with rabbit anti-SP-A antibody, anti-TLR-4 (ab13556, Abcam Inc, Cambridge, MA), anti-TNFR1(sc-374186, Santa Cruz Biotechnology, Dallas, Texas). For cell IF analysis, the cells were fixed with 4% paraformaldehyde, and examined with anti-TLR-4(ab13556, Abcam Inc, Cambridge, MA), anti-TNFR1(sc-374186, Santa Cruz Biotechnology, Dallas, Texas) and anti-Megalin antibody (sc-16478, Santa Cruz Biotechnology, Dallas, Texas) to confirm the types of cultured cells and to determine the purity and quantity of proximal tubular epithelial cells. Slides were stained using Alexa 488 (ab150073, Abcam Inc, Cambridge, MA) and/or Alexa 594-conjugated secondary antibodies (A11058, Life Technologies, Eugene, OR) at room temperature for 1 h for fluorescence visualization of primary antibodies.

### 4.8 Western blotting analysis

Western blot analysis was performed in line with our previous works (8). In brief, lung, kidney tissues, and proximal renal tubular epithelial cells were homogenized in RIPA buffer comprised of a mixture of protease and phosphatase inhibitors (Roche, Indianapolis, IN) in addition to aprotinin (MP Biomedicals, LLC, Illkirch, France). The supernatants were harvested for Western blot analysis following centrifugation at 12000 rpm for 10 min. Total protein concentrations of the lung, kidney samples, proximal renal tubular epithelial cells, and exosomes were determined using a BCA protein assay kit (Thermo Scientific, Rockford, IL). 50μg of total proteins from each sample were resolved through reducing and electrophoresis on 10% or 12% SDS-polyacrylamide gel, followed by transfer onto PVDF membranes (Bio-Rad, Hercules, USA). The blot was blocked using tris-buffered saline containing 5% non-fat milk for 1h and then incubated at 4°C overnight, with a primary antibody against SP-A, or cleaved caspase-3 (#9661, Cell Signaling Technology), or NGAL(sc-515876, Santa Cruz Biotechnology, Dallas, Texas), or cleaved caspase-1(#89332, Cell Signaling Technology), or NLRP3 (# PA5-20838, Thermo Scientific, Rockford, IL), or GSDMD (ab209845, Abcam Inc, Cambridge, MA), or ASC (#67824, Cell Signaling Technology) at 4°C overnight. In this study, β-actin antibody (sc-130657, Santa Cruz Biotech, Dallas, Texas) was used as internal control. The membranes were then incubated using an HRP-conjugated secondary antibody (Bio-Rad, Hercules, CA) and detected with Pierce ECL Western Blotting Substrate (Thermo Scientific, Rockford, IL) and then exposure to X-ray film (Pierce Biochemicals, FL). The expression of the protein was quantified by ImageJ software version 1.48 (Wayne Rasband, NIH, Bethesda, MA). In some experiments, blots were stripped to remove antibodies, through incubation in 2% SDS, 0.06M Tris/HCl (pH 7.0), and 0.72 M 2-mercaptoethanol for 30 min at 25°C, and then reprobed with another primary antibody.

### 4.9 ELISA assay for cytokines

Serum IL-6, TNF-α, and IL-1β levels were assayed with commercially mouse ELISA kits following manufacturer instructions (KMC0061 and KMC3011, Life Technologies, Frederick, MD) (8).

### 4.10 Primary proximal tubular epithelial cells isolation and culture

Primary RTECs from WT and KO mice were isolated in sterile conditions according to previously stated methods with slight modifications (8, 47, 48). Renal cortices were dissected in ice-cold HBSS and sliced into small fragments and then digested in PBS buffer with 1 mg/ml type I collagenase (Worthington, Lakewood, NJ) and 125 μg/ml defined trypsin inhibitor (Gibco) at 37°C for 30 min. The resulting supernatant was sieved through two nylon sieves (pore size: 200 um and 70 μm) and tubular fragments caught by the sieve were flushed in the reverse direction with PBS and centrifuged at 200×g for 5 min. The resulting pellet was resuspended and kept in DMEM/F12 medium containing 5% FBS, 100 IU/ml penicillin, and 100 μg/ml streptomycin, 1×insulin-transferrin-selenium, and 1× MEM nonessential amino acids. Incubation of the plate was done in a humidified incubator under 5% CO_2_ at 37°C and the medium was changed every other day until 90% of cell cultures had been organized as a confluent monolayer.

### 4.11 Isolation, characterization of exosomes from serum

Exosomes were isolated from mouse serum using the Total Exosome Isolation solution (Invitrogen, USA), according to the manufacturer’s instructions. Each serum sample was briefly centrifuged at 2,000×g for 30 min to remove cells and debris. Then 200 μl of total Exosome Isolation solution was added to the 1ml serum, mixed the serum/reagent mixture well by vortexing, and incubated at 4°C for 30min. After centrifugation at 10,000 ×g for 10 min at room temperature, the supernatant was discarded. The pellets containing the exosomes were resuspended in 120μl PBS and then several exosomal surface markers (CD81, CD63, and TSG101) were analyzed by Western blotting. The protein content of exosomes was determined by the Micro-BCA assay kit (Pierce Biotechnology, Rockford, IL, USA). Exosomes diameter detection was performed using a Zetatrac (Microtrac Inc.) instrument, and the area-based mean particle sizes were presented. Zeta potential measurements were carried out on a Malvern Nano-ZS zeta sizer at room temperature (49). The size of the EVs was characterized by scanning electron microscopy.

### 4.12 *In vivo* study of exosomes in WT mice

To evaluate the role of exosomes in the development of AKI, the serum exosomes were used from WT sepsis and sham group mice at 24h after, respectively. In brief, WT mice were injected with exosomes 2 mg/mouse (from about 2ml mouse serum) into the tail vein. The kidney and lung tissues were collected 48 h after the injection. The size of the EVs was characterized by scanning electron microscopy.

### 4.13 Treatment of exosomes in RTECs

RTECs extracted from WT and KO mice were either treated with 50 μg/ml septic-serum exosomes or sham exosomes in serum-free medium for 24 hours. Cells and medium were subsequently harvested for further analysis. RTECs from SP-A KO mice were treated with 50 μg/ml exosomes with or without exogenous SP-A protein (10 μg/ml) for 24 hours. Thereafter, the conditioned media from cultured cells was harvested for the further analysis.

### 4.14 Statistical analysis

Statistical analysis of the data was made using GraphPad Prism software (version 5.0) and presented as mean ± SEM. Comparison among groups was completed by One-way ANOVA or t-test. Animal survival was determined through the Kaplan-Meier survival analysis. For the sake of comparison, *p*< 0.05 was considered statistically significant.

## Data availability statement

The raw data supporting the conclusions of this article will be made available by the authors, without undue reservation.

## Ethics statement

The animal study was reviewed and approved by The Institutional Animal Care and Use Committee of the SUNY Upstate Medical University with protocol #380. Written informed consent was obtained from the owners for the participation of their animals in this study.

## Author contributions

XC performed experiments, analyzed data, interpreted results, and drafted the manuscript. JG, SM, GV, TL, WWX, YX, WCX, OA-R performed partial experiments and editing. GW conceived the study and designed experiments, interpreted results, and wrote the manuscript. All authors contributed to the article and approved the submitted version.
